# Kinetics of Immunolatex Deposition at Abiotic Surfaces under Flow Conditions: Towards Quantitative Agglutination Assays

**DOI:** 10.3390/ijms24010692

**Published:** 2022-12-30

**Authors:** Paulina Żeliszewska, Jolanta Szych, Monika Wasilewska, Zbigniew Adamczyk

**Affiliations:** 1 Jerzy Haber Institute of Catalysis and Surface Chemistry Polish Academy of Science, 30-239 Krakow, Poland; 2 Biomex Co., Ltd., 30-009 Krakow, Poland

**Keywords:** agglutination assays, deposition of immunolatex, electrophoretic mobility of immunolatex, flow cell, immunolatex, zeta potential of immunolatex

## Abstract

Physicochemical properties of immunolatex, prepared by incubation of negatively charged polystyrene microparticles with polyclonal rabbit IgGs, were determined by a variety of experimental techniques. These comprised dynamic light scattering (DLS), laser Doppler velocimetry (LDV) and atomic force microscopy (AFM). The particle diffusion coefficient, the hydrodynamic diameter, the electrophoretic mobility, the zeta potential and the suspension stability were determined as a function of pH for different ionic strengths. The deposition of the immunolatex on bare and polyallylamine (PAH) functionalized mica was investigated using the microfluidic oblique impinging-jet cell, with an in situ, real-time image analysis module. The particle deposition kinetics was acquired by a direct particle enumeration procedure. The measurements enabled us to determine the range of pH where the specific deposition of the immunolatex on these substrates was absent. We argue that the obtained results have practical significance for conducting efficient flow immunoassays governed by specific antigen/antibody interactions.

## 1. Introduction

Protein immobilization at carrier particles of various sizes plays an essential role in enzymatic catalysis, bioreactors, immunological assays, etc. In the case of metal nanoparticle carriers, a controlled protein attachment leads to the corona formation, which is widely studied for single molecule systems and for mixtures comprising the blood serum [1,2,3,4,5,6,7]. However, such systems exhibit a limited stability and low light scattering efficiency that prohibit their wider use for biosensing purposes.

In contrast, immobilization of protein molecules on particles of larger sizes—for example, polymer microspheres (often referred to as latexes)—is advantageous because such conjugates show considerably larger stability compared to the native protein solution [8,9,10]. Therefore, their physicochemical properties and stability can be well-characterized by conventional experimental techniques such as static and dynamic light scattering (DLS), electrophoresis, Laser Doppler Velocimetry (LDV), turbimetry, nephelometry, etc.

A particularly important role is played by polymer microparticles conjugated with appropriate immunoglobulins or antigens, referred to as immunolatexes. Beginning from the pioneering work of Plotz 1956 [11], who developed the first latex agglutination assay for rheumatoid arthritis, such particles are used in a plethora of other tests against various bacterial infections, most often *E. coli*, *Salmonella* [12,13,14,15,16], viral infections such as HIV and, recently, SARS-Cov 2 [17,18,19,20]. The extensive application range of such tests is the effect of their simplicity and short execution time, of the order of minutes [21], which is particularly important for points of care testing.

The tests are performed using micro-liter volumes of the immunolatex suspension, mixed with a similar volume of biological sample containing the targeted antigen. The specific reaction of the antigen with the antibody present in the polymer particles leads to a bulk aggregation of the suspension, which can be observed given the large light scattering efficiency of the microparticles. However, at the expense of simplicity and fast reaction time, most of the currently applied tests can only yield qualitative results. Another limitation stems from the fact that the tests are usually carried out on a glass slide or a similar substrate, inadequately characterized with respect to their surface properties. This may result in interference from the nonspecific deposition of the immunolatex on the substrates. Thus, elimination of this effect could considerably decrease the immunolatex volume needed per test, meaning a decrease in the consumption of immunoglobulins and protein antigens: for example, the receptor-binding domain (RBD) protein of the SARS-Cov2 virus.

However, despite the significance of the non-specific adsorption of immunolatex particles at solid substrates, no systematic studies of this issue have been reported in the literature. Therefore, considering the lack of adequate information, the goal of this work was to elucidate mechanisms of such particle deposition on mica, representing a model negatively charged substrate, and polyallylamine- (PAH) modified mica, representing a positively charged substrate. The experiments were performed in the oblique impinging-jet cell (OBJ) [22], enabling real-time and in situ observations of particle deposition/desorption under well-controlled laminar flow conditions. Using this technique, the non-specific particle attachment to these substrates was thoroughly investigated as a function of pH. It is expected that the acquired information about deposition mechanisms of immunolatex under dynamic conditions can be exploited for the purpose of devising more efficient and quantitative biosensing assays.

## 2. Results and Discussion

### 2.1. Physicochemical Characteristics of Particles

The size distributions of the bare polymer particles (referred to as L800) and the Salmonella immunolatex particles (referred to as SAL particles) were determined by atomic force microscopy (AFM) and by dynamic light scattering (DLS), which furnished the diffusion coefficient of the particles. Using the diffusion coefficient, the particle hydrodynamic diameters, corresponding to their sizes, were calculated using the Stokes–Einstein formula. Compared to AFM, the DLS measurements were more universal, enabling us to determine the dependence of the particle sizes on pH and ionic strength.

**Figure 1:**
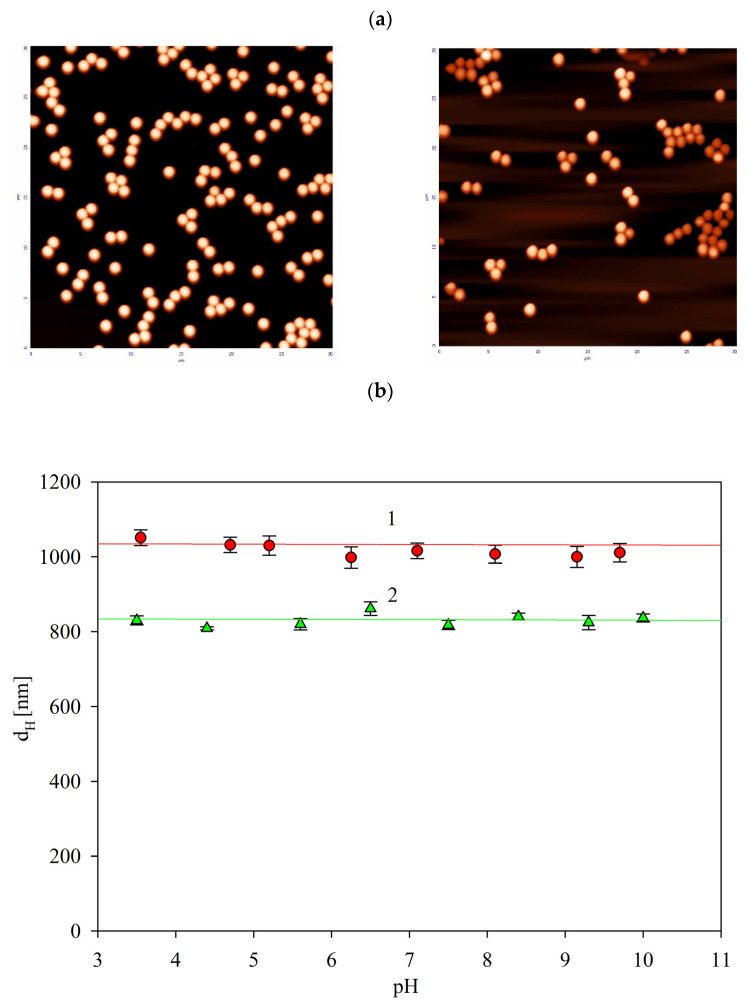
(**a**) The AFM images of the bare polystyrene particles (L800), left hand side, and the salmonella immunolatex particles (SAL), right hand side (scale 30 µm × 30 µm). (**b**) Dependencies of the hydrodynamic diameter of the particles on pH determined by the Stokes–Einstein equation using the DLS diffusion coefficient data, 1 mM NaCl. 1. SAL particles, bulk concentration 100 mg L^−1^; 2. L800 particles, bulk concentration 100 mg L^−1^. The lines are guides for the eyes.

The particle layers imaged by AFM are shown in Figure 1a. The average particle sizes determined by the procedure described in Experimental Methods were equal to 820 ± 40 and 840 ± 30 nm for the L800 and the SAL samples, respectively. The increase in the immunolatex particle size was caused by the presence of the IgG molecule corona, whose characteristic dimension (hydrodynamic diameter) is equal to 12 nm [23].

The hydrodynamic diameter data, derived from DLS and shown in Figure 1b, indicate that the L800 particle size was independent of pH for the range of 3 to 10, with average values equal to 830 ± 20 nm, similar to that determined by AFM. On the other hand, the DLS size of the SAL particles was slightly larger, equal to 1030 ± 30 nm for the pH range 3 to 10. The increase in the particle size was the result of their reversible association, which led to the increase in the hydrodynamic resistance coefficient and, as a consequence, to the increase in the hydrodynamic diameter calculated by the Stokes equation. Hence, the results shown in Figure 1b confirm an adequate stability of the particles for this broad pH range.

**Figure 2:**
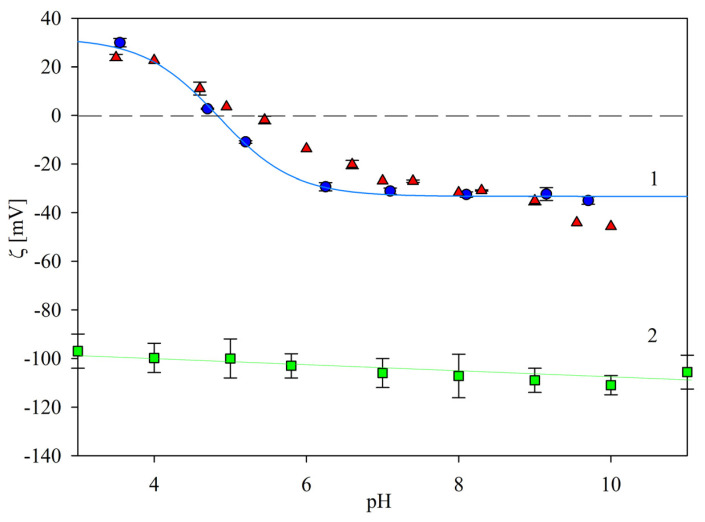
Dependencies of the zeta potential of the immunolatexes on pH, 10 mM NaCl; (●) results for the SAL immunolatex particles in this work; solid line 1 shows the interpolation of these results. (▲): Previous results for the mouse monoclonal IgG coated immunolatex without BSA blocking, Ref. [24]. Solid line 2 shows the interpolation of the bare latex results.

Electrokinetic characteristics of the particles were acquired according to the procedure described in Experimental Methods. Primarily, the electrophoretic mobility was measured for different ionic strengths as a function of pH using the laser Doppler velocimetry (LDV) method. Next, the zeta potential was calculated using the Smoluchowski equation. The results obtained for 10 and 1 mM NaCl ionic strength are presented in Figure 2. As can be seen, the zeta potential of the bare L800 particles was equal to −97 ± 5 mV at pH 3.5, and slightly decreased to −110 ± 5 mV at pH 7.4 (for the 10 mM NaCl concentration). In contrast, the zeta potential of the immunolatex SAL particles was positive for a pH below 5.5, assuming the maximum value of 30 ± 3 mV at pH 3.5 (for the 10 mM NaCl). At a pH higher than 6, the zeta potential of the immunolatex assumed a negative value of −30 ± 3 mV, which was constant for a pH of up to 10. It is interesting to mention that the dependence of the SAL particle zeta potential on pH practically matched the previously determined dependence pertinent to the mouse monoclonal IgG functionalized latex particles [24].

**Figure 3:**
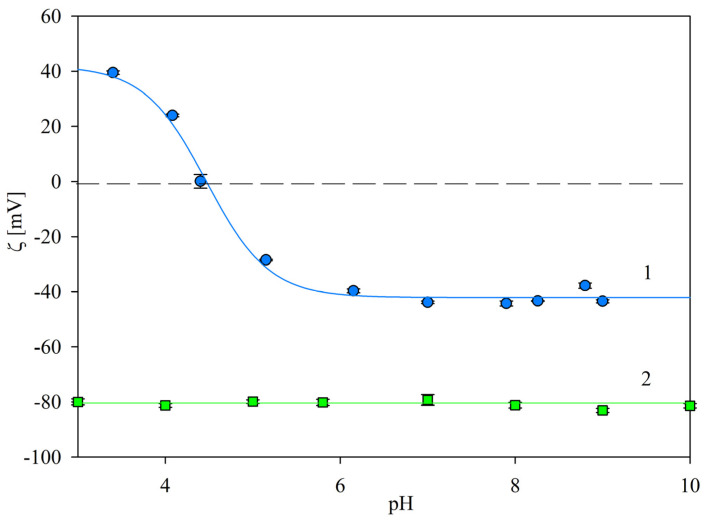
Dependence of the zeta potential of the SAL immunolatex particles on pH, 1 mM NaCl. (●): Experimental results derived from LDV (solid line 1 shows the interpolation of these results). Solid line 2 shows the interpolation of the bare latex results.

Analogous results were obtained for the lower ionic strength of 1 mM NaCl; see Figure 3. In this case, the zeta potential of the SAL immunolatex particles was positive for a pH below 5, assuming the maximum value of 40 ± 4 mV at pH 3.5, became negative at a pH above 5 and then rapidly decreased to −45 ± 5 mV at pH 7.4.

**Figure 4:**
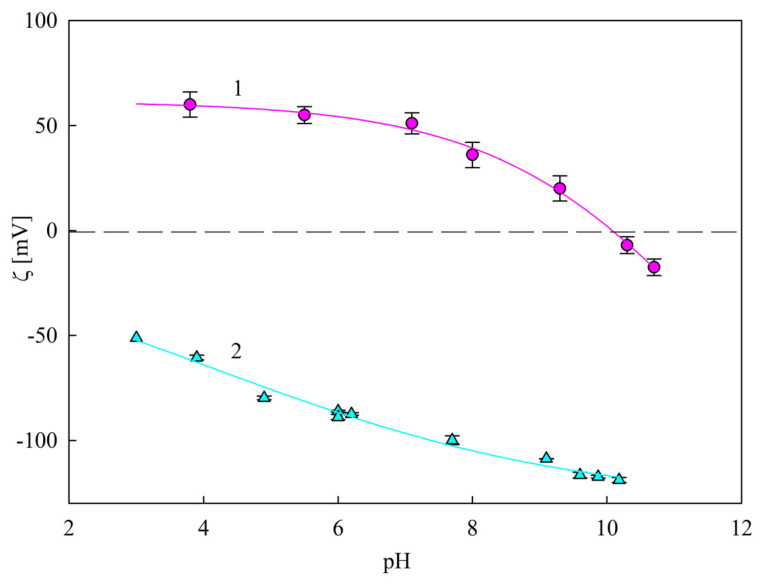
Dependence of the zeta potential of mica on pH, determined by the streaming potential method. 1—(●) mica/PAH [25]; 2—(▲) bare mica, 1 mM NaCl. The solid lines represent fits of experimental data.

Additional series of experiments were performed with the aim to determine the immunolatex particle stability over a prolonged storage time. Accordingly, the hydrodynamic diameter and the zeta potential of particles in the 100 mg L^−1^ suspension were determined for prescribed time intervals. It was established that these parameters did not change within a time period of up to 3 months. This was interpreted as adequate stability of the immunolatex suspension, and enabled us to perform reliable deposition kinetic experiments for bare and PAH-modified mica. The electrokinetic characteristics of these substrates were acquired by streaming potential measurements, as described in the Section 3.2. In Figure 4, the dependencies of the substrate zeta potential (calculated using the Smoluchowski formula) on pH are shown. In the case of bare mica, the zeta potential decreased from −50 mV at pH 3.5 to −100 mV at pH 7.4 (for 1 mM NaCl concentration). For the PAH-modified mica, the zeta potential remained positive for a pH up to 9, and was equal to 55 and 35 mV at pH 3.5 and 7.4, respectively.

### 2.2. Deposition Kinetics of the Immunolatex Particles

The deposition kinetic experiments were carried out in a microfluidic OBIJ cell (Experimental Methods), which facilitated both in situ and real-time observation of the deposited particles using optical microscopy. The aim of this series of experiments was to determine the pH ranges in which the non-specific SAL immunolatex particle deposition at bare or PAH modified mica due to electrostatic-type interactions was absent. Such results can serve as reliable controls for agglutination assays, where the particle deposition is driven by the specific antigen/antibody interactions.

**Figure 5:**
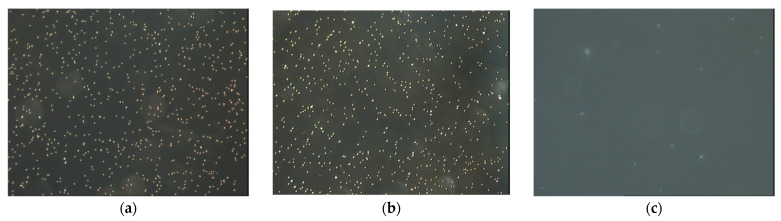
Micrographs of the SAL immunolatex particles on mica acquired in situ by optical microscopy; 1 mM NaCl, deposition time 120 min, micrograph size: 135 µm × 100 µm. (**a**) pH = 3.5, (**b**) pH = 4, (c) pH = 5–7.4.

Micrographs of the SAL immunolatex particles on mica, taken for various pHs after the deposition time of 120 min, are shown in Figure 5. One can observe that the particle density abruptly decreased with pH, becoming negligible for a pH of 5. The deposition kinetics can be quantified by introducing the surface concentration of particles (defined, for the sake of convenience, as the number of particles per unit area of the substrate, hereafter referred to as $N_{p}$), as was previously carried out for the investigation of microparticle deposition [24,26,27] and protein adsorption [28].

**Figure 6:**
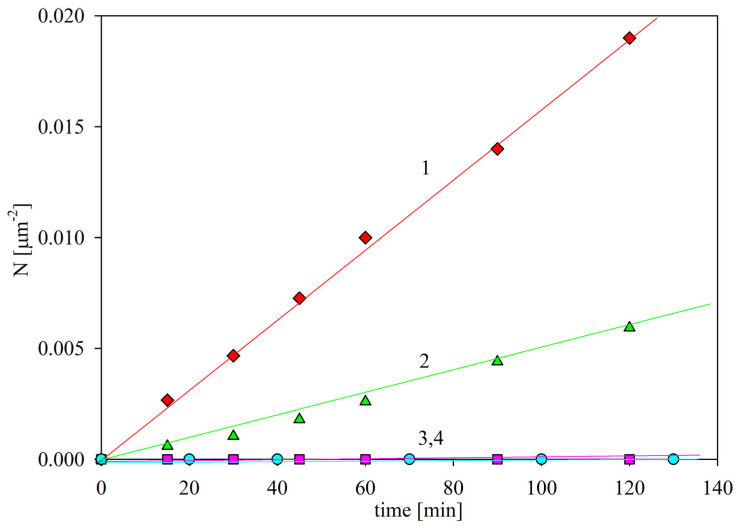
The kinetics of SAL immunolatex particle deposition in bare mica in the OBIJ microfluidic cell, shown as the dependence of the surface concentration on the deposition time. Particle bulk con-centration, 100 mg L^−1^, 1 mM NaCl; flow rate 2.5 × 10^−3^ cm^3^ s^−1^. The points show the results obtained for: 1. pH 3.5 (♦); 2. pH 4 (▲); 3. pH 5.5 (■) and 4. pH 7.4 (●). The solid lines are linear fits of the experimental data.

The SAL particle deposition kinetics of bare mica, acquired for the bulk suspension concentration of 100 mg L^−1^ (this corresponds to 0.01 % mass concentration), 1 mM NaCl, flow rate 2.5 × 10^−3^ cm^3^ s^−1^ and various pHs, is shown in Figure 6. One can observe that at a pH of 3.5, the particle surface concentration linearly increases with time, which can be described by the formula:

(1) $$N_{p}=k_{c}c_{b}t$$

where *k_c_* is the mass transfer rate constant in the cell, $c_{b}$ is the particle bulk concentration and *t* is the deposition time.

The experimental mass transfer rate constant at pH 3.5, denoted as *k_c_*_0_, was equal to 1.7 × 10^−6^ L (mg min)^−1^ µm^−2^. This maximum mass transfer constant is used as the reference value for calibration of the results obtained for other pHs.

For higher pHs, the SAL particle deposition rate in mica abruptly decreased (see Figure 6); accordingly, for pH 4, the mass transfer rate constant was equal to 5.0 × 10^−7^ L (mg min)^−1^ µm^−2^; and for pH 5.5 to 7.4, the particle deposition was negligible. It is interesting to note that this behavior correlates with the decrease in the zeta potential of the SAL particles (see Figure 3), which was equal to 40 and 25 mV at pH 3.5 and 4, respectively, whereas the zeta potential of the mica substrate varied between −50 and −60 mV for this pH range. At a pH higher than 5, the zeta potential of the particles was negative, i.e., of the same sign as the zeta potential of mica. The strict correlation of the particle deposition rate with the zeta potential indicates that it was governed by the electrostatic interactions, as is analogous with colloid particle behavior [27]. This is an important conclusion, suggesting that non-specific immunolatex interactions with surfaces can be predicted without performing tedious deposition kinetic investigations if the mean-field zeta potentials of the particles and the substrate are known.

**Figure 7:**
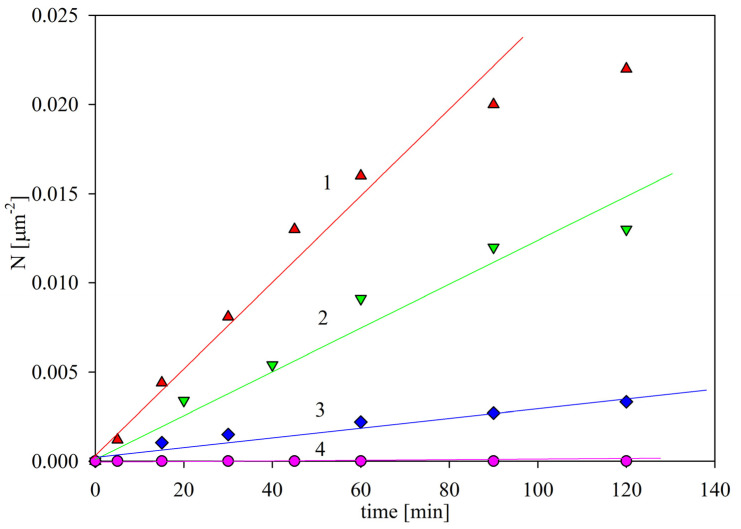
The kinetics of SAL immunolatex particle deposition at PAH-modified mica under convection conditions (OBIJ microfluidic cell ) shown as the dependence of the surface concentration on the deposition time; particle bulk concentration 100 mg L^−1^, 1 mM NaCl; flow rate 2.5 × 10^−3^ cm^3^ s^−1^. The points show the results obtained for: 1. (▲) pH 9–7.4; 2. (▼) pH 7; 3. (♦) pH 6; 4. (●) pH 5–3.5. The solid lines are linear fits of the experimental data.

In order to further test this hypothesis, the SAL particle deposition kinetics on PAH-modified mica exhibiting positive zeta potential for a pH of up to 9 (see Figure 4) was investigated. Representative results obtained for various pHs and 1 mM NaCl concentration are shown in Figure 7. As can be seen, at pH 7.4–9, the particle deposition rate was at its maximum, and was characterized by the mass transfer rate constant of 2.0 × 10^−6^ L (mg min)^−1^ µm^−2^, similar to that which was previously determined for bare mica. For lower pHs, the adsorption kinetics abruptly decreased, and were characterized by a mass transfer rate constant of 1.2 × 10^−6^ and 2.0 × 10^−7^ L (mg min)^−1^ µm^−2^ for pH 7 and 6, respectively. At a pH higher than 5, the deposition rate became negligible. Similarly as for bare mica, the decrease in the deposition efficiency correlated with the zeta potential of the SAL particles, which became positive at a pH lower than 5, i.e., of the same sign as the zeta potential of PAH-covered mica, equal to 55 mV.

Given that most of the assays were carried out at pH values ranging between 7.4 and 8 [13,14,29,30,31], these results indicate that a significant interference from the non-specific particle deposition can appear for substrates that exhibit a positive surface charge (zeta potential.)

The results obtained for negatively and positively charged substrates can be uniquely analyzed by introducing the scaled particle deposition efficiency, defined as follows:

(2) $$\bar{k}=k_{c}(pH)/k_{c0}$$

where $k_{c}(pH)$ is the mass transfer rate, experimentally determined for a fixed pH as the slope of the linear dependence of *N_p_* on the deposition time.

**Figure 8:**
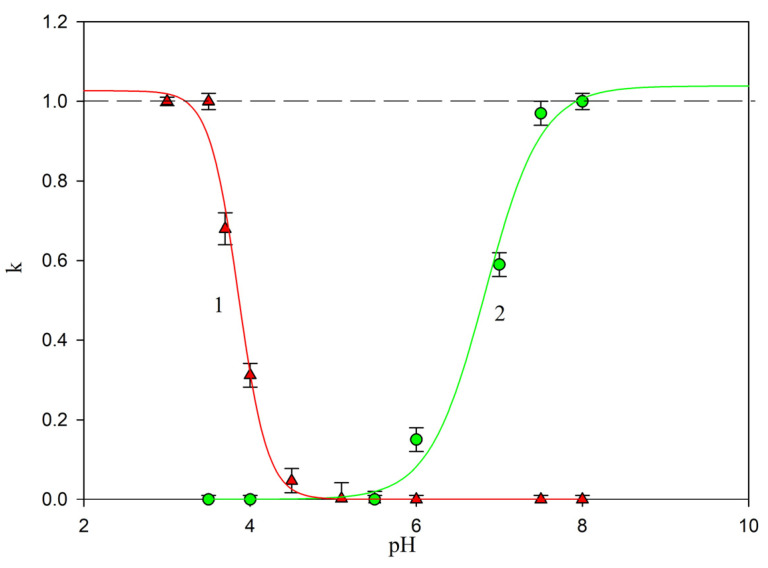
Normalized rate of SAL immunolatex particle deposition on bare mica (▲) and PAH-modified mica (●) as a function of pH, 1 mM NaCl. Solid lines 1 and 2 represent a fit of experimental data for bare and PAH-covered mica, respectively.

Experimental results transformed using Equation (2) are shown in Figure 8. It can clearly be seen that the non-specific deposition of the SAL immunolatex particles in negatively charged mica vanishes at pH > 4, i.e., at a pH above their isoelectric point. On the other hand, for positively charged substrates, the non-specific deposition appears at a pH of above 6, i.e., the typical pH range occurring during the performance of latex agglutination tests.

## 3. Materials and Methods

### 3.1. Materials

The suspension of negatively charged sulfonate polystyrene microparticles used as colloid carriers for antibodies, hereafter referred to as L800, was our own product, synthesized according to the Goodwin procedure [32]. The stock suspension of the concentration, determined by densitometry and the dry mass method, was diluted to 100 mg L^−1^ in the DLS and LDV measurements.

Salmonella Immunlatex was a commercial product of Biomex ( Krakow, Poland) prepared by non-covalent coupling of the L800 latex particles (with the bulk concentration of 10,000 mg L^−1^) with the anti- rabbit polyclonal antibodies.

The SAL particle suspension was cleaned before each deposition experiment by a thorough membrane filtration.

Water was purified using a Milipore Elix 5 apparatus. Chemical reagents (sodium chloride, hydrochloric acid) and Polyallylimine PAH, were purchased from Sigma Aldrich Merck KGaA, Darmstadt, Germany. The average molar mass of the PAH sample of 70 kDa was more precisely determined by dynamic viscosity measurements [33].

Ruby muscovite mica, obtained from Continental Trade, was used as a model substrate. The solid pieces of mica were freshly cleaved into thin sheets prior to each particle deposition experiment, which were carried out in a diffusion cell under thermostated conditions.

The modification of the mica substrate by PAH was carried out according to the previously described procedure [22]. Briefly, a few freshly cleaved mica sheets were vertically immersed in the PAH solution of a controlled concentration, pH and ionic strength, typically equal to 10 mg L^−1^, 5.6 and 10^−3^ mol L^−1^, respectively. The adsorption was continued under pure diffusion conditions for 10 minutes in a thermostated cell. Afterward, modified mica was washed three times in water and placed on the flow cell.

### 3.2. Experimental Methods

The diffusion coefficient of bare and immunolatex particles (hereafter referred to as SAL particles) was determined by dynamic light scattering (DLS) using the Zetasizer Nano ZS instrument from Malvern (A.P. Instruments, Warsow, Poland). The hydrodynamic diameter was calculated using the Stokes–Einstein relationship. The electrophoretic mobility of bare and immunolatex was measured using the Laser Doppler Velocimetry (LDV) technique, using the same apparatus. The zeta potential was calculated using the Smoluchowski formula.

The zeta potential of bare and PAH-modified mica sheets was determined by the streaming potential method, using a microfluidic cell in the form of the parallel plate channel [22]. Initially, the streaming potential was measured using a pair of reversible electrodes as a function of the hydrostatic pressure difference Δ*P*. Subsequently, the streaming potential was converted to the zeta potential *ζ* using the Smoluchowski relationship [34].

Atomic force microscopy (AFM) measurements were carried out using the NT-MDT OLYMPUS IX71 device with the SMENA scanning head. The measurements were performed in semi-contact mode, using silicon probes and polysilicon cantilevers HA-NC ETALON, with resonance frequencies of 140 kHz +/−10% or 235 kHz +/−10%.

The deposition kinetics of the particles was investigated using the oblique impinging jet cell, according to the previously described procedure [22]. A steady laminar flow of the suspension was generated by the peristaltic pump, which enabled us to regulate the volumetric flow rate within broad limits. It should be mentioned that because of the under-pressure prevailing in the cell, the mica substrate, in the form of freshly cleaved sheets, was firmly attached to the cell wall without using any adhesive. This eliminated the possibility of contamination of the cell during the measurement.

Deposited particles were observed in situ using optical microscope equipped with long-distance objectives, camera and imaging processing software. The number of particles per unit area (typically one square micrometer, denoted hereafter by *N_p_*) was determined by a direct counting of over 10–20 equal sized areas randomly chosen over the mica surfaces. This provides a relative precision of these measurements of more than 2%. The temperature during the experiments was kept at a constant value, equal to 298 ± 0.1 K.

## 4. Conclusions

Despite the significance of the non-specific adsorption of immunolatex particles in solid substrates, especially in the convection controlled regime, no systematic studies of this issue have been carried out in the literature. Considering the lack of adequate information, thorough kinetic experiments were performed using the oblique impinging-jet cell, thus enabling real time and in situ observations of particle deposition/desorption events. Using this technique, the non-specific particle attachment was determined as a function of pH.

It was established that for the substrate exhibiting negative zeta potential (bare mica), the deposition kinetics of particles vanished at a pH above 5. This result has practical significance, confirming that at a pH range of 7.4–8, at which most tests are carried out, the non-specific deposition of the particles on negatively charged surfaces becomes negligible.

On the other hand, for the substrates exhibiting positive zeta potential (mica/PAH), a significant deposition of the immunolatex particles was observed at a pH above 6, which suggests that such substrates are not appropriate for performing latex agglutination assays.

These results are also significant for basic science, because they confirm that immunolatex particle deposition is governed by electrostatic interactions, depending on the zeta potential of the substrates and particles. Thus, determination of of physicochemical characteristics of immunolatex particles, comprising their electrophoretic mobility and the zeta potential as a function of pH, makes it possible to predict their interactions with abiotic surfaces when performing agglutination assays.

It is expected that the obtained results can be exploited to efficiently perform reliable, label-free immunological assays under flow conditions, creating the possibility of direct detection of deposited particles via optical imaging.

## Data Availability

The data are available upon request.

## References

[1] Monopoli M.P., Aberg C., Salvati A., Dawson K.A. (2012). Biomolecular coronas provide the biological identity of nanosized materials. Nat. Nanotechnol..

[2] Winzen S., Schoettler S., Baier G., Rosenauer C., Mailaender V., Landfestera K., Mohr K. (2015). Complementary Analysis of the Hard and Soft Protein Corona: Sample Preparation Critically Effects Corona Composition. Nanoscale.

[3] Lee Y.K., Choi E.J., Webster T.J., Kim S.H., Khang D. (2015). Effect of the protein corona on nanoparticles for modulating cytotoxicity and immunotoxicity. Int. J. Nanomed..

[4] Baimanov D., Cai R., Chen C. (2019). Understanding the Chemical Nature of Nanoparticle-Protein Interactions. Bioconjugate Chem..

[5] Zeng L., Gao J., Liu Y., Gao J., Yao L., Yang X., Liu X., He B., Hu L., Shi J. (2019). Role of protein corona in the biological effect of nanomaterials: Investigating methods. Trends Anal. Chem..

[6] Wang X., Zhang W. (2022). The Janus of Protein Corona on nanoparticles for tumor targeting, immunotherapy and diagnosis. J. Control. Release.

[7] Khan S., Sharifi M., Gleghorn J.P., Babadaei M.M.N., Bloukh S.H., Edis Z., Amin M., Bai Q., ten Hagen T.L.M., Falahati M. (2022). Artificial engineering of the protein corona at bio-nano interfaces for improved cancer-targeted nanotherapy. J. Control. Release.

[8] Martin-Rodriguez A., Ortega-Vinuesa J.L., Hidalgo-Alvarez R. (2002). Electrokinetics of Protein-coated Latex Particles, International Electrokinetics and Electrophoresis. Surf. Sci. Ser..

[9] Kawaguchi H., Ohshima H. (2016). Latex Diagnosis, Encyclopedia of Biocolloid and Biointerface Science 2V Set.

[10] Gosecka M., Chehimi M.M., Basinska T., Słomkowski S. (2017). Adsorption and covalent binding of fibrinogen as a method for probing the chemical composition of poly(styrene/α-tert-butoxy-ω-vinylbenzyl-polyglycidol) microsphere surfaces. Colloids Surf. B Biointerfaces.

[11] Plotz C.M., Singer J.M. (1956). The latex fixation test. I. Application to the serologic diagnosis of rheumatoid arthritis. Am. J. Med..

[12] Ristaino P.A., Levine M.M., Young C.R. (1983). Improved GM1-enzyme-linked immunosorbent assay for detection of *Escherichia coli* heat-labile enterotoxin. J. Clin. Microbiol..

[13] Luz D., Shiga E.A., Chen G., Quintilio W., Andrade F.B., Maranhão A.Q., Caetano B.A., Mitsunari T., Silva M.A., Rocha L.B. (2018). Structural Changes in Stx1 Engineering Monoclonal Antibody Improves Its Functionality as Diagnostic Tool for a Rapid Latex Agglutination Test. Antibodies.

[14] Silva M.A., Santos A.R.R., Rocha L.B., Caetano B.A., Mitsunari T., Santos L.I., Polatto J.M., Horton D.S.P.Q., Guth B.E.C., Dos Santos L.F. (2019). Development and Validation of Shiga Toxin-Producing *Escherichia coli* Immunodiagnostic Assay. Microorganisms.

[15] Piazza R.M., Caetano B.A., Henrique C.P., Luz D., Munhoz D.D., Polatto J.M., Rocha L.B., Silva M.A., Mitsunari T., Charles S., Gurtler P.V. (2020). Chapter 6—Immunological tests for diarrhoea caused by diarrhoeagenic *Escherichia coli* targeting their main virulence factors. Methods in Microbiology.

[16] Shiga E.A., Guth B.E.C., Piazza R.M.F., Luz D. (2020). Comparative analysis of rapid agglutination latex test using single-chain antibody fragments (scFv) versus the gold standard Vero cell assay for Shiga toxin (Stx) detection. J. Microbiol. Methods.

[17] Esmail S., Knauer M.J., Abdoh H., Voss C., Chin-Yee B., Stogios P., Seitova A., Hutchinson A., Yusifov F., Skarina T. (2021). Rapid and accurate agglutination-based testing for SARS-CoV-2 antibodies. Cell Rep. Methods.

[18] Whitman J.D., Hiatt J., Mowery C.T., Shy B.R., Yu R., Yamamoto T.N., Rathore U., Goldgof G.M., Whitty C., Woo J.M. (2020). Test performance evaluation of SARS-CoV-2 serological assays. Nat. Biotechnol..

[19] Li Z., Yi Y., Luo X., Xiong N., Liu Y., Li S., Sun R., Wang Y., Hu B., Chen W. (2020). Development and clinical application of a rapid IgM-IgG combined antibody test for SARS-CoV-2 infection diagnosis. J. Med. Virol..

[20] Peeling R.W., Wedderburn C.J., Garcia P.J., Boeras D., Fongwen N., Nkengasong J., Sall A., Tanuri A., Heymann D.L. (2020). Serology testing in the COVID-19 pandemic response. Lancet Infect. Dis..

[21] Paek S.H., Lee S.H., Cho J.H., Kim Y.S. (2000). Development of rapid one-step immunochromatographic assay. Methods.

[22] Żeliszewska P., Wasilewska M., Cieśla M., Adamczyk Z. (2021). Deposition of Polymer Particles with Fibrinogen Corona at Abiotic Surfaces under Flow Conditions. Molecules.

[23] Dąbkowska M., Adamczyk Z. (2014). Mechanism of immunoglobulin G adsorption on mica-AFM and electrokinetic studies. Colloids Surf. B Biointerfaces.

[24] Żeliszewska P., Wasilewska M., Adamczyk Z. (2017). Monolayers of immunoglobulin G on polystyrene microparticles and their interactions with human serum albumin. J. Colloid Interface Sci..

[25] Morga M., Adamczyk Z. (2013). Monolayers of cationic polyelectrolytes on mica--electrokinetic studies. J Colloid Interface Sci..

[26] Yu Y.S., Wang M.C., Huang X. (2017). Evaporative deposition of polystyrene microparticles on PDMS surface. Sci. Rep..

[27] Adamczyk Z., Morga M., Nattich-Rak M., Sadowska M. (2022). Nanoparticle and bioparticle deposition kinetics. Adv. Colloid Interface Sci..

[28] Wasilewska M., Adamczyk Z., Sadowska M., Boulmedais F., Cieśla M. (2019). Mechanisms of Fibrinogen Adsorption on Silica Sensors at Various pHs: Experiments and Theoretical Modeling. Langmuir.

[29] Peruski A.H., Peruski L.F. (2003). Immunological methods for detection and identification of infectious disease and biological warfare agents. Clin. Diagn. Lab. Immunol..

[30] Darwish I.A. (2006). Immunoassay Methods and their Applications in Pharmaceutical Analysis: Basic Methodology and Recent Advances. Int. J. Biomed. Sci..

[31] Li P., Deng S., Zech Xu Z. (2021). Toxicant substitutes in immunological assays for mycotoxins detection: A mini review. Food Chem..

[32] Goodwin J., Hearn J., Ho C.C., Otewill R.H. (1974). Studies on the preparation and characterisation of monodisperse polystyrene laticee. Colloid Polym. Sci..

[33] Jachimska B., Jasiński T., Warszyński P., Adamczyk Z. (2010). Conformations of Poly(allylamine hydrochloride) in Electrolyte Solutions: Experimental Measurements and Theoretical Modeling. Colloids Surf. A.

[34] von Smoluchowski M. (1903). Contribution to the Theory of Electro-Osmosis and Related Phenomena. Bull. Int. L’Academie Sci. Crac..

